# One Size Does Not Fit All: Contextualising Family Physical Activity Using a Write, Draw, Show and Tell Approach

**DOI:** 10.3390/children4070059

**Published:** 2017-07-14

**Authors:** Robert J. Noonan, Stuart J. Fairclough, Zoe R. Knowles, Lynne M. Boddy

**Affiliations:** 1 Department of Sport and Physical Activity, Edge Hill University, Ormskirk L39 4QP, UK; Stuart.fairclough@edgehill.ac.uk; 2 Physical Activity Exchange, Research Institute for Sport and Exercise Sciences, Liverpool John Moores University, Liverpool L3 2AB, UK; Zoe.R.Knowles@ljmu.ac.uk (Z.R.K.); Lynne.M.Boddy@ljmu.ac.uk (L.M.B.); 3 Department of Physical Education and Sport Sciences, University of Limerick, Limerick V94 T9PX, Ireland

**Keywords:** physical activity, children, family, accelerometer, ActiGraph, diary, raw, context, write, draw, show and tell (WDST)

## Abstract

Understanding family physical activity (PA) behaviour is essential for designing effective family-based PA interventions. However, effective approaches to capture the perceptions and “lived experiences” of families are not yet well established. The aims of the study were to: (1) demonstrate how a “write, draw, show and tell” (WDST) methodological approach can be appropriate to family-based PA research, and (2) present two distinct family case studies to provide insights into the habitual PA behaviour and experiences of a nuclear and single-parent family. Six participants (including two “target” children aged 9–11 years, two mothers and two siblings aged 6–8 years) from two families were purposefully selected to take part in the study, based on their family structure. Participants completed a paper-based PA diary and wore an ActiGraph GT9X accelerometer on their left wrist for up to 10 weekdays and 16 weekend days. A range of WDST tasks were then undertaken by each family to offer contextual insight into their family-based PA. The selected families participated in different levels and modes of PA, and reported contrasting leisure opportunities and experiences. These novel findings encourage researchers to tailor family-based PA intervention programmes to the characteristics of the family.

## 1. Introduction

Regular physical activity (PA) provides school-age children (referred to as “children” herein) with broad-ranging physical and psychological health benefits [1,2]. The United Kingdom (U.K.) Chief Medical Officers recommend that children accumulate at least 1 hour of moderate-to-vigorous PA (MVPA) each day, and minimise time spent in sedentary behaviours to achieve and maintain health [3]. However, few U.K. children currently meet these recommended guidelines [4].

Independent of a child’s age and gender, parents are among the strongest of influences on a child’s PA [5], influencing their child’s PA level and mode (e.g., outdoor play, organised sport and active travel) via the support they provide (e.g., logistical support, verbal encouragement and praise) [6,7] and their parenting style [8,9,10]. The structure [11,12,13] and socioeconomic status (SES) of the family [14] often determines the parental support and parenting style that children receive. For example, Lareau [15] found that mid- to high-SES parents engaged their children in a process of “concerted cultivation” by way of enrolling them in a broad range of organised activities, whereas low-SES parents facilitated the process of “natural growth” through supporting and encouraging unstructured activities, including outdoor play. To date, there is limited U.K. research that explores the influence of family structure on children’s PA, especially with regards to the opportunities and restrictions they experience. Further research comparing the PA behaviours and experiences of children from contrasting family structures, such as nuclear and single-parent families, may help inform future targeted interventions to increase children’s PA in specific population subgroups based on family characteristics and modes of participation.

Family-based PA interventions are considered an effective way to increase children’s [16] and parents’ PA [17,18]. Family-based PA interventions that engage with families in a formative sense prior to intervention delivery and are tailored to the characteristics, motivations and time constraints of the family have been shown to be most effective [16]. However, little evidence exists on effective ways in which to engage families in PA research. Family-based PA research poses unique methodological challenges to researchers who must contend with whole-family recruitment and family-level data collection [19]. Prior to designing PA intervention programmes for families, it is important to understand their current PA behaviour.

Accelerometers are the most widely used objective measure of PA, providing a direct assessment of frequency, intensity and duration, but limited contextual understanding of activities undertaken [20]. Moreover, they are unable to capture water-based (i.e., swimming) and non-ambulatory activities, which can bias objective PA estimates [21,22] and provide limited understanding as to why some families are more active than others. Focus groups offer an effective way of extending, validating and contextualising objective data collected through quantitative methods, and provide a unique person-centred insight into factors that influence individual behaviour [23]. Focus groups have been used to explore children’s [6,24] and parents’ PA perceptions and experiences [25,26], but few studies have included whole family units (i.e., children *and* their parents [27,28]). The dynamics of a family focus group differ greatly to those of a traditional focus group. Family focus groups require more interactive, inclusive and developmentally appropriate methods to equally engage children and parents in the focus group and limit socially desirable responses from participants in the presence of each other [29].

The “write, draw, show and tell” (WDST) methodology is an evolution of the focus group and the write/draw method [6]. WDST adopts a holistic child-centred humanistic philosophy. It views children as experts and gives them opportunity to voice their perceptions and lived experiences in different ways, to minimise adult/researcher influence or bias. WDST is particularly suited for research with children for reasons of inclusivity and interactivity [30,31]. The combination of methods and subsequent triangulation of multiple data sources allows for the topic to be analysed from several angles, thereby enhancing data credibility and trustworthiness, and strengthening the evidence of the phenomenon under investigation [32,33]. Although the WDST methodology was specifically developed for use with children, we believe it may also be appropriate for family-based PA research.

Methodological knowledge on conducting research with whole families, and suitable techniques to assess family PA, perspectives and lived experiences would help support researchers and practitioners to conduct research *with* rather than *on* families. Therefore, the aims of the study were to: (1) demonstrate how a WDST methodological approach can be appropriate to family-based PA research, and (2) present two distinct family case studies to provide insights into the habitual PA behaviour and experiences of a nuclear and single-parent family. For the purpose of this study, the Smith family was considered to be representative of a single-parent family, and the Jones family was considered to be representative of a nuclear family. A key purpose of this study was to demonstrate the utility of combining methods when undertaking research *with* families. In this study, we demonstrate the value of the participant “voice”, and encourage researchers to contextualise objective PA data to illuminate and make sense of family PA behaviour. Although the findings of the study are based upon the PA behaviours and experiences of two distinct families, we see the proposed methods having applicability and scalability to larger family-based research projects. Moreover, we see the proposed methods being of use to researchers in other health-related fields, such as social work and family-based therapy, and consider them transferable to real-world settings to assist practitioners engaged in health promotion and formative work with families.

## 2. Materials and Methods

### 2.1. Participants

Two families comprising of a “target” child aged 9–11 years, sibling aged 6–8 years, and mother were recruited through primary schools in Liverpool, U. K. The families were purposefully selected and invited to take part in the present study based on their demographic and PA data collected as part of a larger study (see [34]). The families were selected to represent diverse family structures and contrasting PA behaviour patterns to demonstrate the limitations of a “one-size-fits-all” approach to family-based PA promotion and intervention programming. All parents and children gave written informed consent and assent to take part. The study received institutional ethics approval (reference number: 15/SPS/023), and data collection took place between June 2015 and April 2016. Each family received a £60 high street shopping voucher in return for their participation in the project.

### 2.2. Measures

A baseline parent questionnaire ascertained marital status (married, widowed/divorced/separated, single and never married, or living with partner); employment status (1–16 h, 17–30 h, or >30 h); typical working hours (Monday–Friday, weekends, and shift patterns); relationship to child (mother, father, guardian, or other); and number, sex and age of siblings living at home. Parents also reported whether they had access to a self-contained garden (yes or no). Household distance to school was objectively measured using the Google Maps online route planner (Googleplex, Mountain View, CA, USA, https://www.google.co.uk/maps) to estimate the shortest route from school addresses to parents’ reported home addresses [35].

#### 2.2.1. Socioeconomic Status

Parents’ reported home postcodes were imported into the GeoConvert (http://geoconvert.mimas.ac.uk/) application [36] to calculate area-level SES based on the English indices of multiple deprivation (IMD) 2015. The IMD is a U.K. Government-produced measure comprising seven areas of deprivation (income, employment, health, education, housing, environment, and crime). Individual-level SES was assessed using the highest level of parent education for each family. Responses included high school, college, university, and higher-degree education [37].

#### 2.2.2. Anthropometrics

Stature and body mass were taken at home addresses for all participants by the first author using standard procedures [38]. Body mass index (BMI) was calculated from height and weight (kg/m²), and BMI cut-points were used to classify children’s [39] and adults’ weight status [40].

#### 2.2.3. Physical Activity

PA methods have been reported in full elsewhere (see [34]). Briefly, participants completed a paper-based PA diary and wore an ActiGraph GT9X (ActiGraph, Pensacola, FL, USA) accelerometer on their left wrist during waking hours for seven consecutive days. They were instructed to only remove the monitor during water-based activities and when sleeping. Accelerometers were collected from home addresses after the seven measurement days, the data was downloaded, and they were then returned to participants on the following Friday to be worn on weekend days on three occasions. This process was repeated in the subsequent season, resulting in a total of 10 weekday and 16 weekend measurement days per participant. One family (Smith family) completed measures throughout June/July (summer) and November/December (autumn/winter) 2015, and the other (Jones family) completed measures throughout October/November (autumn) 2015 and March/April (spring) 2016. ActiGraph data were downloaded using ActiLife version 6.11.4 (ActiGraph, Pensacola, FL, USA), converted to raw CSV format and processed in R (http://cran.r-project.org) package GGIR (version 1.2-0, https://cran.r-project.org/web/packages/GGIR/) to classify the time spent in MVPA using the Euclidean Norm Minus One (ENMO) method [41,42]. ActiGraph raw data wear times were estimated on the basis of the standard deviation and value range of each axis, calculated for 60 min moving windows with 15 min increments [42]. A time window was classified as non-wear time if, for at least two out of the three axes, the standard deviation was less than 13.0 mg, or if the value range was less than 50 mg [42]. A valid day was classified as 10 h or more of accelerometer wear. Wrist-worn specific ActiGraph equations provided by Hildebrand et al. [41] were used to classify MVPA. The Hildebrand equations were solved for three metabolic equivalents of task (METs), resulting in MVPA cut-points of 201.4 mg and 100.6 mg for children and mothers, respectively. The mean MVPA for each participant was calculated for weekdays (Monday–Friday) and weekends (Saturday–Sunday). The weekend MVPA was then averaged over four weekends for phases 1 and 2. Diary data were analysed thematically. Responses to each diary category—mode (e.g., football, walking) and duration of activity (in minutes), start and end times, location of activity and with whom the activity was undertaken (e.g., “on my own”, “with friend”, “with brother/sister”)—were summed to produce frequency counts. These were subsequently expressed as a percentage of the total number of entries for each participant, and averaged for each phase of the data collection.

#### 2.2.4. Focus Groups

Family focus groups were arranged and conducted by the first author. A degree of trust and rapport had been established between the researcher and the families prior to the focus groups due to the participants’ involvement in the research programme for the previous 12 months, whereby the researcher regularly liaised with the families and collected anthropometric and accelerometer data [34]. Parents were sent a text message by the first author to arrange a mutually convenient time for the focus group. Prior to conducing the focus groups, the first author provided each family with a pack containing a write/draw booklet, coloured pencils, and task instructions. For the purpose of this study, children were instructed to draw the front cover of a book that showed them taking part in an out-of-school activity that they enjoyed. This write/draw activity would later serve as an elicitation activity during the focus group. The home provided a suitable setting for the focus group, as it served as a safe and familiar location for participants and removed transportation barriers. Both focus groups were conducted in the living room of the family home.

Semi-structured focus group guides informed by the WDST framework (see [6] and Table 1 for detail) were used to ensure consistency across each focus group. Participants were provided with self-adhesive note paper, a clip board and a pencil. Participants were encouraged to write down on paper their responses to questions, and in their own time, provide a verbal account to their written responses. This provided participants with greater control over their expression, and offered time to articulate their own meanings embedded within their written responses. By providing children and parents with interactive ways of sharing their perceptions and experiences, we anticipated that this would facilitate more open discussion, limit social desirable responses, and thus elicit more representative and detailed perceptions on family PA that may otherwise have remained uncovered when using traditional focus group approaches [6]. Focus groups were audio-recorded using a digital recorder, and were transcribed verbatim for further analysis and anonymised. Two focus groups were conducted, each comprising a mother and two children, lasting approximately 30 min (mean = 28.7), resulting in 48 pages of raw transcription data (Arial font, size 12, double spaced; Microsoft, Redmond, WA, USA).

### 2.3. Data Management and Analysis

The focus group generated visual (write/draw and show/tell activity) and narrative data (show/tell activity and children’s write/draw narratives). The separate data sources were pooled together and a mixed analysis approach was taken for complimentary purposes. For the WDST data, children’s narratives were transcribed verbatim, classified as a written “report”, and subsequently appended to visual data for each participant. The reports and visual data were used in combination to categorise “marks” on paper in relation to specific themes (i.e., PA mode, parent support; see [6] for detail). The narrative data were analysed via thematic content analysis. After listening to the focus group recordings and reviewing the transcripts, the first author generated a series of overarching themes aligned to the aims of the study [43]. The two transcripts were then analysed comparatively to identify similar and contrasting themes, and were further explored to seek understanding for these differences. To ensure accuracy and facilitate alternative interpretations of the data, the focus group recordings, transcripts and drawings were independently reviewed by the third author, and were then cross-examined against the data in reverse, from the themes to the data sheets.

## 3. Results

A total of six participants from two families participated. This included two target children (boy, *n* = 2), two siblings (boy, *n* = 1) and two mothers. All participants were of healthy weight and white ethnic origin. They lived in lower-than-average-SES neighbourhoods, reflected by their high IMD scores (27.5 (tertile 4) and 36.6 (tertile 5), compared to the English average of 23.64 (tertile 4) [44]. Accelerometer data for each family and diary data for each child participant is presented before the narrative data. A case description of each family is provided below in Boxes 1 and 2 to offer insight into the context and background of each family. Pseudonyms were assigned to families and individual participants to assure anonymity.

### Box 1: Jones Family

The Jones family is a nuclear family. The family comprises a mother, father and two male children. Joseph and Matthew were aged 10 and 6, respectively. The family home is situated in a suburban neighbourhood (IMD of 27.5; tertile 4) with access to a self-contained garden. Both parents are employed and degree-educated. Mum and dad work part-time and full-time across weekdays, respectively. The school is in an affluent area of the city (12.0; tertile 2), 1.1 km from home. The children do not travel to school actively. The family has access to two cars.

### Box 2: Smith Family

The Smith family is a single-parent family. The family comprises a mother and four children (Tom, aged 10; Sophie, aged 8; Paul, aged 4; and Chloe, aged 2). The family home is a terraced house situated in an urban residential area (IMD of 36.6; tertile 5) with no access to a self-contained garden. Mum is unemployed. The school is in a deprived area of the city (38.4; tertile 5), 1.4 km from home. The children walk to and from school daily. The family does not have access to a car.

**Figure 1:**
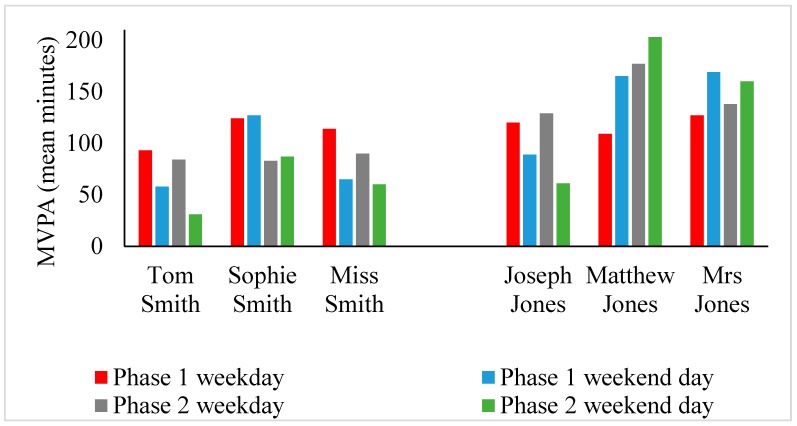
Mean weekday and weekend-day moderate-to-vigorous physical activity (MVPA) family comparisons for each participant and phase.

**Figure 2:**
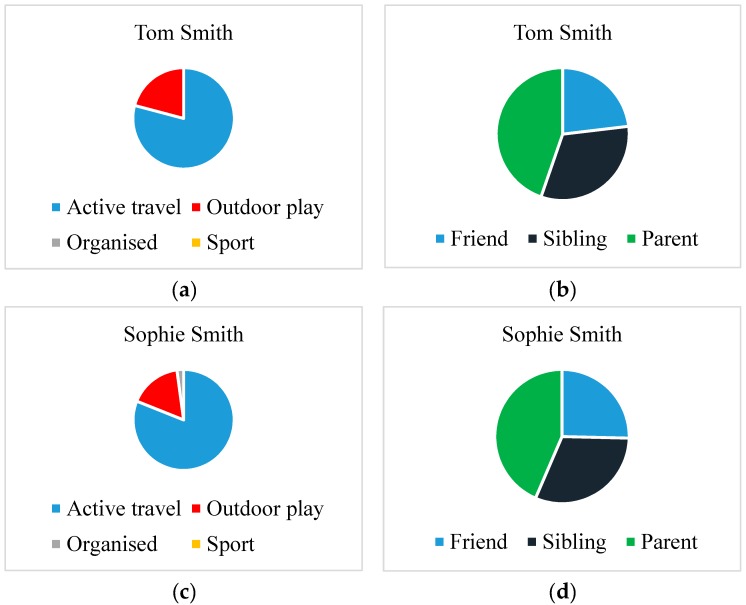
(**a**,**b**) Physical activity (PA) diary data for Tom Smith, and (**c**,**d**) PA diary data for Sophie Smith.

Figure 1 presents the median weekday and weekend-day MVPA levels for each participant by data collection phase, and Figure 2 presents the overall PA diary data for the child participants. There was a combined total of 117 recorded entries for Tom (*n* = 31), Sophie (*n* = 34), Joseph (*n* = 21), and Matthew (*n* = 31). Tom’s and Sophie’s PAs involved active travel and outdoor play, and were mostly undertaken with friends (Figure 1a–d). Their active-travel levels were consistent across phases, but their outdoor play levels were higher in phase 1 than in phase 2 (see supplementary file S1). Joseph and Matthew reported no participation in active travel and only some parent–child PA (Figure 2a–d). This parent–child activity took place on weekend days. Most of their PA took place with friends at sports club settings, especially on weekdays (see supplementary file S1).

## 4. Discussion

In this study, we introduce the WDST methodology for use with families and provide a practical checklist and considerations for future applications. Below we describe the experience of conducting family focus groups using the WDST methodology to elicit the habitual PA behaviour and experiences of a nuclear and a single-parent family. We highlight that while challenging, family focus groups generate rich contextual family-level data, providing great insight into family behaviour patterns, processes and experiences. The themes identified in the data are subsequently presented. 

The focus group gave families the opportunity to add context to their accelerometer data. To date, mostly objective data has been used to characterise children’s [45], parents’ [46], and parent–child PA [47,48,49]. It became evident in the focus group that some of the objective PA estimates may have been biased due to legitimate monitor non-wear and the inability of the accelerometers to capture water-based (i.e., swimming) and cycling activities [21,22]. For example, according to the accelerometer data, Joseph Jones recorded lower MVPA levels on weekends compared to weekdays, yet this pattern of activity was not consistent with the diary or focus group data. The focus group data exposed that Joseph removed the accelerometer during sport participation for safety reasons, which would have provided an underestimation of his typical MVPA level during these time periods.

*“Because he’s a goalie he can’t obviously wear it, with the gloves. It’s good that we haven’t got it [accelerometer] on this week. He’s found his bike again. Also, when we go swimming, you have to take it off”*.

[Mrs. Jones]

*“You should get them waterproof”*.

[Joseph Jones]

This finding demonstrates the value of diaries to validate accelerometer data. Other studies have demonstrated the utility of using diaries in combination with accelerometers to promote accelerometer wear [50,51] and describe the context of PA [52,53]. Here we extend beyond these studies by introducing an accelerometer, diary and narrative-based approach to more accurately quantify and contextualise habitual PA among families. Using the themes identified in the data, PA-level, mode and location, parental support and style, and family-based PA, we demonstrate below the value and importance of assessing PA context (i.e., location, mode, other participants) when conducting research *with* families.

### 4.1. Physical Activity Level, Mode and Location

**Figure 3:**
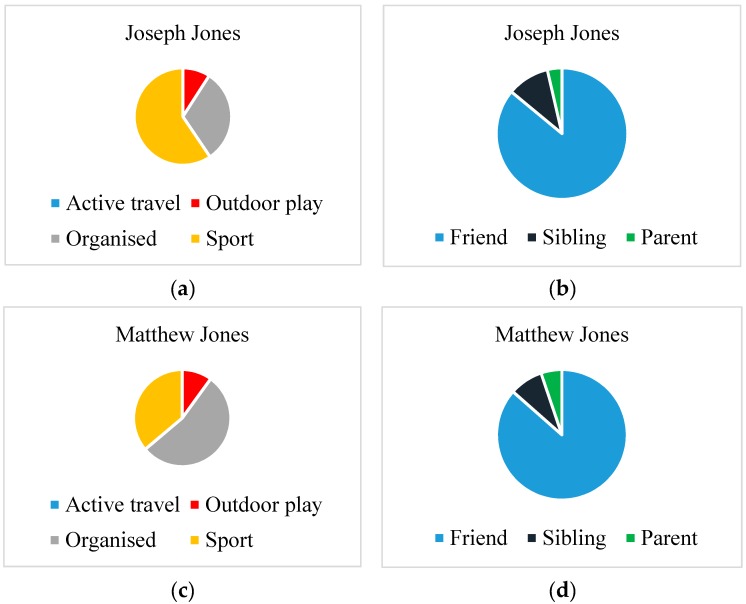
(**a**,**b**) PA diary data for Joseph Jones, and (**c**,**d**) PA diary data for Matthew Jones.

**Figure 4:**
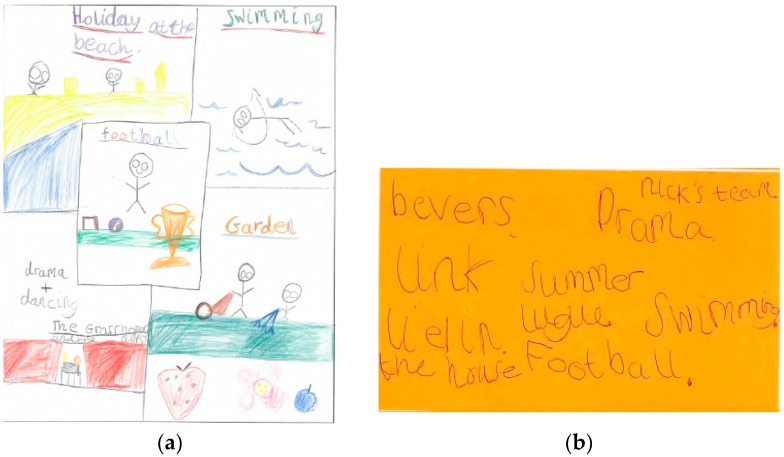
(**a**,**b**) Write, draw, show and tell (WDST) data for Matthew Jones, illustrating leisure activities. *“Well, holiday. When I went to Malaga in Spain. When I went to Mummy's cousin's. Making sandcastles and jumping on the waves. I enjoy swimming. And this is me in the garden. Daddy's doing the wall, and I'm doing where you cut the grass at the side. And this one here is me playing football for my team. And drama and dance. We've just done a show. I was Adam Ant”* [Matthew Jones].

The families in this study participated in different levels (Figure 1) and modes of PA (Figure 2a,c and Figure 3a,c). The Smith children participated in outdoor play and active travel, whereas the Jones children participated in organised activities and sport. The out-of-school activities of the Jones children were highly structured both on weekdays and weekend days. The children did not walk or cycle to school, but engaged in a broad range of after-school activities on most weekdays. These activities took place at regular times on specific days for each week of measurement. Alongside football, the Jones children undertook other structured activities (e.g., Beaver scouts—i.e., a club focussed on outdoor skills, such as camping and adventurous activities—and drama). These activities are depicted in Matthew’s WDST data below (Figure 4a,b).

**Figure 5:**
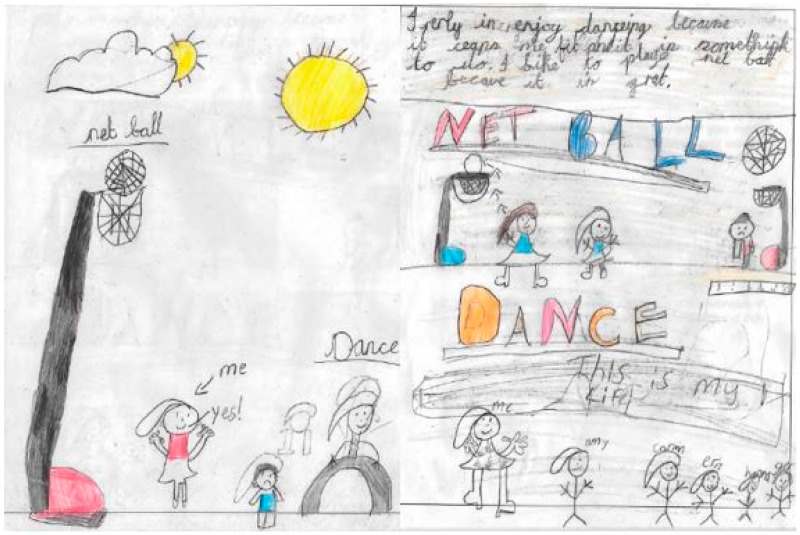
Drawing from Sophie, aged 8, illustrating out-of-school PA. *“In my drawing, I’ve got me, I’ve got my friends, and I’ve got things at the park. I really enjoy street dance because it’s something I do. And then netball, I like to play netball because it is great. Sometimes at after school clubs, sometimes Miss picks us to go in the front, but sometimes you can go at the back. But I’ve been in the front. But I’m mainly at the back”* [Sophie Smith].

**Figure 6:**
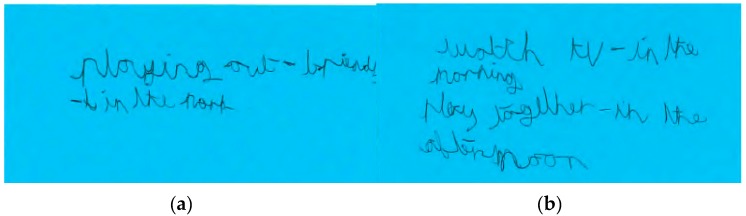
(**a**,**b**) Show/tell data illustrating Tom Smith’s leisure activities. *“Playing out. I’ve been playing out with my friends in the park. My friends from school. We just walk everywhere we go. Straight after school I would either just go to the park with my mates or go to the forest. The one by school or the one by Mackie’s, the rec. And if I don’t do that, then I'll go home and get ready. Get changed, and then out to the park. I'm allowed like to the rails and back. I’m allowed to stay there til 8 o’clock. I tend to just stay in on weekends”* [Tom Smith].

In contrast, the Smith children walked to and from school on weekdays and played outdoors after school in the neighbourhood with their school friends. Their MVPA levels were lower on weekend days compared to weekdays. The decline in MVPA between weekdays and weekend days was greater for Tom than Sophie (Figure 1). Sophie reported participating in more structured after-school activities (e.g., netball and dance) than Tom on weekdays, and spent more time with her mother actively commuting on weekend days. These activities were depicted in her drawing (Figure 5). This was contrary to her brother who spent most of his weekend time indoors playing video games and watching TV (Figure 6a,b).

Although all children recounted at least some experience of unstructured PA, the locations within which these experiences took place differed between the families. For example, the Smith children played outdoors in the neighbourhood, whereas the Jones children played within the confines of the home. For the Jones children, the family garden served as a key resource for their PA. They have access to activity equipment including a basketball net and football goal and use the equipment regularly, especially in the summer months. Interestingly, the diary and narrative data revealed a seasonal decline in outdoor play, but not in active travel. Previous research by Harrison et al. [54] found no association between season and active travel to school. Perhaps this is because for some children there is no access to a family car, and thus travel to school actively independent of weather conditions. On the other hand, the decline in ambient light after school in the autumn/winter months can heighten parents’ neighbourhood safety concerns, and in turn, reduces the independence children are given to play outdoors [55,56]. Indeed, Tom reported a reduction in his independent mobility during the winter months, and noted that he was less motivated to play outdoors with his friends in the winter months as it was “*colder and wetter”,* and instead preferred to remain indoors. Tom, Sophie and Miss Smith configured their narratives about outdoor play in these terms:

*“I wouldn’t really play out as long or as much in the winter because it’ll be too cold and wet to play out. We usually just stay at home because it’s too cold to go anywhere”*.

[Tom Smith]

*“If it was winter, we just stay in and have the fire on all the time. Cocoa, and watch films”*.

[Sophie Smith]

*“Yes, and you wouldn’t be allowed out as late, would you? We’re pretty lazy in the winter, aren’t we?”*.

[Miss Smith]

Based on these findings, children’s PA intervention programmes may be best suited to the after-school period in the autumn and winter seasons. Further research is warranted to examine seasonal variation in specific activity modes, such as active travel, outdoor play and organised sport. Future studies investigating seasonal variation in PA should consider the adoption of diaries to capture activities otherwise not captured by accelerometers. Using accelerometers alone may underestimate seasonal differences in PA [21].

Differences in PA modes were also evident between the parents. Most of Mrs Jones’s PA was gym-based and took place on weekends when off work. Intuitively, she recorded more MVPA on weekend days compared to weekend days. Miss Smith on the other hand accumulated all her activity through active travel and household chores, most of which was recorded on weekdays when she walked to and from school with her children. Based on this finding, consideration should be given to childcare, household and occupational responsibilities when designing family-based PA intervention programmes, as it was evident, both in this study and others, that such factors are key barriers to parents’ [57] and family-based PA [7,58].

*“Well, mine aren’t very fun, but walking and the big obvious one is cleaning. I don't enjoy it, but it is a good form of exercise”*.

[Miss Smith]

### 4.2. Parental Support and Style

The types of support the children received from their parent(s) differed between the two families. The Jones children received support through actions, such as logistic and financial support, whereas the Smith children experienced more verbal encouragement and co-participation. These findings are broadly consistent with the work of Brockman et al. [14], who found that parental support for children’s PA differed by family SES. We also found that parenting styles differed between the two families, in respect to both enabling and restricting children’s PA. It was evident from the narrative data that parenting styles influenced the children’s mode of PA and their spatial PA patterns. The Jones children experienced what Lareau [15] describes as “concerted cultivation”. They were enrolled in a broad range of out-of-school activities and received limited independent mobility to play outdoors.

*“We don’t want him hanging round the streets. That’s why we do all these activities, because we said yesterday, that’s fine going from A to B, that’s not a problem, but he wants to go from A to wherever, on the bike, and it’s not happening. Dad said no matter what age, he still needs to keep contact. He wants to know where they are 24 hours a day”*.

[Mrs Jones]

This was quite the opposite to the out-of-school experiences of the Smith children. The Smith children received a high level of independent mobility from their mother. She placed some spatial and temporal boundaries on their outdoor play. For example, Miss Smith reported that “*Tom must return home by 8 o’clock and is not allowed to travel further than the local park*”. These boundaries were firmer in the winter months when it was “*darker*”, as Miss Smith perceived the neighbourhood to be *“less safe”* compared to in the summer and spring months. Sophie, the younger of the two Smith children, received less independent mobility compared to her brother, Tom.

*“You wouldn’t be allowed out as late in the winter, would you? She’s allowed to play out from the corner to the little green box. That's the distance she’s allowed to go, with her being smaller”*.

[Miss Smith]

### 4.3. Family-Based Physical Activity

Identifying strategies to actively engage parents in family-based PA programmes is a priority for PA research and practice. A potential way of engaging parents in family-based PA intervention programmes is to encourage parent–child PA [17,59]. In this study, the prevalence of parent–child PA differed between the two families. The Smith family engaged in parent–child PA by way of walking to and from school on weekdays, and to public spaces (e.g., the local park) on weekend days.

*“We’ve been to the park today for a little kick about and had another big walk round”*.

[Miss Smith]

However, the Jones family reported limited parent–child PA. In contrast, the Jones children undertook most of their PA with friends at sport clubs (Figure 3b,d), whereas their parents’ PA was mainly undertaken at the health club. Interestingly, the Jones children reported spending time with their parents, but this was in the context of travelling to and from structured activities, rather than engaging in PA with their parents.

*“Dad takes Joseph the majority of the time, and I sort Matthew, or I take them both. I go to the gym in the village”*.

[Mrs Jones]

**Figure 7:**
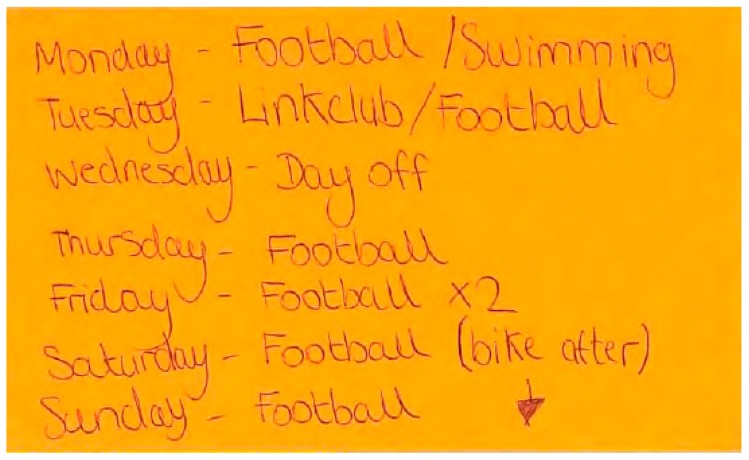
Show/tell data illustrating Joseph Jones’ structured leisure activities.

Another interesting finding was that the Jones children’s high enrolment in structured activities influenced their parents’ PA levels. At the time of the focus group, Joseph was attending football training sessions most days of the week (Figure 7), which impacted on the frequency with which his mother was able to visit the gym.

*“Dad will go to the gym straight from work, but I can’t because I’m taking them there. So, I’m frustrated at the moment that I can’t go to the gym at the minute, because I was going to the gym while they were in school on my days off. Because he’s in the winter league and a summer league they’re going to have a match on a Saturday and Sunday. We’ll go to football then we’ll go to church”* [Mrs Jones].

Together, these findings demonstrate the potential adverse effect children’s structured activities can have on parent and parent–child PA, and provide further support for contextualising accelerometer data in family-based research.

Common family-based unstructured activities included walking and visiting public parks/green spaces. Walking for recreation and transport is a low-cost health-enhancing activity that may serve as an effective family-based PA intervention strategy to increase PA among children and parents via the social support they provide each other [60,61]. The Smith family undertook a lot of walking both for recreation and transport purposes. This activity generally took place close to home.

*“We go from school, walk home, get changed, back up to dance, and I either go to my Dad’s or go through the rail, and then back to pick her back up at six, and then back home again to do the tea”*.

[Miss Smith]

Interestingly, none of the Jones children’s or their parents’ PA was attributed to walking. All their activities were organised and located outside the immediate neighbourhood, beyond feasible walking and cycling distance. Thus, the family cars played an important facilitating role in the Jones family’s leisure activities. For example, the health club and football training ground are located over 7 and 15 km away from home, respectively. Previous research has shown that family car ownership is inversely associated with children’s walking levels [62,63]. Interestingly, the family car was also used for short-travel distances, including the home-to-school commute (1.1 km). This could have been due to parental safety concerns, but may also have been in response to the family’s travel habits [64]. Walking serves as an opportunity for children and adults to increase and maintain their daily PA levels without depending on school-related and organised activities or logistic support [65,66,67]. Providing families with guidance on how they can best incorporate these low-cost activities into their schedules, and how unstructured activities contribute to achieving daily PA recommendations and good health, is warranted. Furthermore, this finding provides additional support for mode-specific family-based PA intervention programmes.

With regard to intervention programme timing, participants in this study suggested that the weekend would be the most suitable time to promote family-based PA. The weekend provided these selected families with more opportunity for family-based PA, compared to weekdays, due to children not attending school and parents having fewer work responsibilities. The identification of appropriate intervention timings could be established during the formative phase of intervention programmes when consulting with families. The participants in this study reported that the frequency of family-based outdoor activities declined during the autumn/winter months, relative to the summer/spring months. Therefore, thought may also need to be given to the time of year that family-based PA intervention programmes are delivered.

The presentation of WDST data alongside the accelerometer and diary data demonstrated the utility of mixed-methods research when investigating family PA behaviour. The triangulated data sources showed that the social circumstances of the family play a key role in the activities they undertake. Indeed, some families (e.g., Jones family) are in a more advantageous position than others, both financially (i.e., to pay for club subscriptions and equipment, drive children to places to be active) and environmentally (e.g., access to garden/backyard) to support and foster an active lifestyle. Such rich contextual information would have remained uncovered had we limited our analyses to questionnaire, diary or accelerometer data alone. Based on these findings, it is important that future family-based PA interventions are directed towards promoting mode-specific PA, and tailored to the characteristics of the family. The WDST methodology provides researchers and practitioners with an interactive and inclusive way of eliciting the perceptions and experiences of families during the research process, and may have utility in the formative phase of family-based interventions.

### 4.4. Strengths and Limitations

This study demonstrated the utility of the WDST methodology for use with families and provided a practical checklist and considerations for future application. The study was innovative by way of its mixed-methods design. The combination of accelerometer, diary, write/draw, and narrative provided a unique data set that enabled the exploration of habitual PA behaviour among families in relation to their family characteristics, neighbourhood environment and transport resources. Another unique aspect of this study was the concurrent objective assessment of children’s and parents’ MVPA over 10 weekdays and 16 weekend days. Moreover, the use of diaries revealed an understanding of the mode of PA in which parents and children engaged, and whether they undertook PA together or separately. One limitation of our study was the use of a retrospective narrative to gain understanding of family PA behaviour and experiences. Ecological momentary assessment (EMA) collects momentary self-reports in situ via electronic diaries on smartphones and tablets [68]. This innovative method would facilitate the collection of ecological real-life contextual data on family-based PA to underpin subsequent family-based PA intervention programmes [69]. In addition, we acknowledge that the families in this study were an active homogenous group that are unlikely to be the target group for PA intervention programmes. However, in comparing the two families and highlighting the differences in habitual PA behaviours and experiences, it was our aim to demonstrate the need for mode-specific family-based PA intervention programmes based on family characteristics.

## 5. Conclusions

Using children’s and mothers’ recounted experiences and perceptions of family-based PA, this study demonstrates how the WDST methodological approach can be advantageous when compared to more traditional singular method-based approaches, and provides evidence to support the use of WDST with families. The combination of methods revealed interconnected and complementary findings on family-based PA that would have been overlooked using surveys, diaries, accelerometers or focus groups alone. The families in this study participated in different levels and modes of PA, and reported contrasting leisure opportunities and experiences. By offering voice via the PA narratives of two distinct families, we have highlighted the limitations of the one-size-fits-all approach to family-based PA promotion and intervention programming. These findings encourage researchers to tailor family-based PA intervention programmes to the characteristics of the family. Moreover, the study demonstrates the utility of PA diaries in conjunction with accelerometers to provide context to objectively measured PA levels.

## Figures and Tables

**Table 1:** Write, draw, show and tell (WDST) methodology and considerations.

**Recruitment and Research Process**	Emphasise study benefits that extend beyond physical health benefits (i.e., weight reduction). Offer monetary/tangible incentives to families for participating in the research. Build trustful relationships with families through continual communication and dialogue throughout project using appropriate mechanisms such as text messaging, social media or email. Obtain informed parental consent and child assent.
**Physical activity (PA) Observation**	Objective assessment of PA using wrist-worn accelerometers to boost monitor wear. Subjective assessment of PA using diary to contextualise accelerometer data. Demonstrate study equipment (i.e., accelerometers) and provide verbal and written instructions.
**Focus Group**	Coordinate focus group time with parent emphasising desire for whole-family participation. Conduct at family home address. Circular seating arrangement with researcher sat among family. Researcher and participants address each other by first name. Focus group process including purpose, confidentiality, and right to withdraw provided at beginning of focus group. Individualised feedback on PA status provided.
**Show/Tell**	Participants provided with self-adhesive note paper, clip board and a pencil to write down responses to questions. Participants encouraged to provide a verbal account of the meaning behind written responses. Begin with simple tasks and questions that participants can answer as experts, such as favourite physical activities, interests and likes.
**Write/Draw**	Write/draw activity. Drawing materials (i.e., booklet and coloured pencils) and instructions provided. Children engaged in child-centred informal conversation to verify interpretation and add context to drawing.
**Show/Tell**	More cognitively challenging open-ended questions asked. Questions tailored to the interests and likes of the participants. Age-appropriate terms and terminology used. Ensure children and parents have equal opportunity to contribute by directing specific questions at individual participants. Demonstrate genuine interest in participants’ perspectives (i.e., maintain eye contact, paraphrase responses, relate responses to earlier comment or to that made by another family member). Seek clarification (i.e., probe for deeper explanations and real-life examples).

## Supplementary Materials

The following are available online at www.mdpi.com/2227-9067/4/7/59/s1, Figure S1: Physical activity diary data for weekdays and weekend days for each participant and data collection phase.
