# Supplementary material for: One Size Does Not Fit All: Contextualising Family Physical Activity Using a Write, Draw, Show and Tell Approach
Source: Children (Basel). 2017 Jul 14;4(7):59. doi: 10.3390/children4070059 (PMC5532551; doi:10.3390/children4070059)
Supplement: Supplementary file 1 [file children-04-00059-s001.docx]

Tom Smith physical activity diary data for weekday and weekend days.

Sophie Smith physical activity diary data for weekday and weekend days.

Joseph Jones physical activity diary data for weekday and weekend days.

Matthew Jones physical activity diary data for weekday and weekend days.
